# Individuals With Chronic Kidney Disease Qualifying for SGLT-2 Inhibitors in the United States and EMPA-KIDNEY Generalizability

**DOI:** 10.1016/j.jacadv.2023.100349

**Published:** 2023-05-24

**Authors:** Nicholas Chiu, Rahul Aggarwal, Leonard Chiu, Muthiah Vaduganathan, Gregg C. Fonarow, Deepak L. Bhatt

The EMPA-KIDNEY (Study of Heart and Kidney Protection with Empagliflozin) trial^1^ is the latest in a series of 4 randomized controlled trials of sodium-glucose cotransporter-2 (SGLT-2) inhibitors in patients with chronic kidney disease (CKD)^1^, ^2^, ^3^, ^4^ showing outcome benefits. EMPA-KIDNEY was a global randomized controlled trial of empagliflozin in patients with CKD. Building on previous trials, EMPA-KIDNEY evaluated the broadest range of CKD patients, enrolling patients who had an estimated glomerular filtration rate (eGFR) down to ≥20 mL/min/1.73 m^2^, and also extending to patients with and without diabetes at risk for CKD progression. With EMPA-KIDNEY showing a benefit in composite primary outcome for progression of kidney disease in such a wide range of CKD patients, SGLT-2 inhibitors are likely to be indicated for a broader CKD population in the United States.

We estimated a benchmark for national implementation by determining the number of U.S. individuals eligible for SGLT-2 based on the 4 major trial eligibility criteria. We further characterized the breakdown of individuals eligible for empagliflozin by Kidney Disease Improving Global Outcomes classification.

EMPA-KIDNEY enrollment criteria were applied to the National Health and Nutrition Examination Survey (NHANES) (2009-2018). NHANES is designed to represent the U.S. population by using multistage, stratified, clustered samples of the civilian noninstitutionalized population. NHANES was approved by the National Center for Health Statistics Institutional Review Board.

We included nonpregnant individuals ≥18 years with an eGFR of ≥20 but <45 mL/min/1.73 m^2^ regardless of level of albuminuria or eGFR of ≥45 but <90 mL/min/1.73 m^2^ with a urine albumin-creatinine ratio (UACR) of ≥200 mg/g. Patients were required to be treated with a renin-angiotensin system inhibitor. We excluded those with type 2 diabetes mellitus who had eGFR >60 mL/min/1.73 m^2^ and prior atherosclerotic cardiovascular disease, defined as history of myocardial infarction, angina, or stroke. We further excluded patients with systolic blood pressure <90 mmHg or >180 mmHg.

CREDENCE (Canagliflozin and Renal Events in Diabetes with Established Nephropathy Clinical Evaluation) trial, DAPA-CKD (Dapagliflozin and Prevention of Adverse Outcomes in Chronic Kidney Disease) trial, and SCORED (Effect of Sotagliflozin on Cardiovascular and Renal Events in Patients with Type 2 Diabetes and Moderate Renal Impairment Who Are at Cardiovascular Risk) trial enrollment criteria were also separately applied.^2^, ^3^, ^4^ Finally, the total number of U.S. individuals meeting any of the 4 trial criteria was estimated. Analyses were conducted with R 4.0.5 and survey-weighting design was used to determine national projections.

EMPA-KIDNEY trial criteria applied to 2,951,404 (2,554,433-3,348,374) individuals in the U.S., SCORED applied to 2,834,935 (2,496,276-3,173,593) individuals, DAPA-CKD applied to 1,626,098 (1,356,610-1,895,587) individuals, and CREDENCE applied to 599,296 (444,509-754,083) individuals. Overall, a total of 5,621,804 (5,099,550-6,144,057) individuals were eligible for SGLT-2 inhibition by any of the 4 trial criteria.

**Figure 1:**
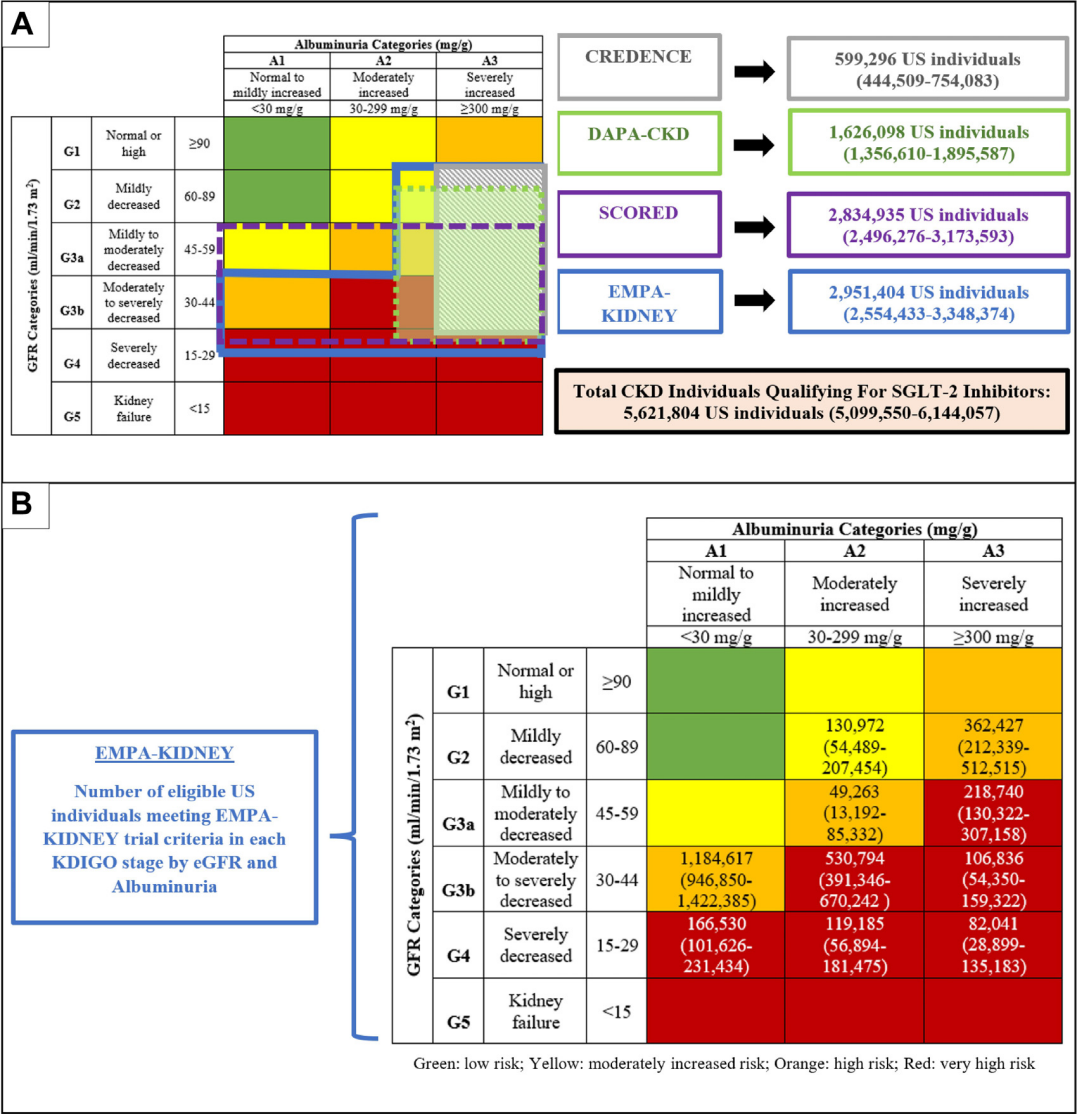
Individuals With Chronic Kidney Disease Qualifying for SGLT-2 Inhibitors in the United States **(A)** U.S. individuals qualifying for SGLT-2 inhibitors by CREDENCE, DAPA-CKD, SCORED, and EMPA-KIDNEY criteria; **(B)** EMPA-KIDNEY eligible individuals based on CKD staging. Estimates presented with 95% confidence intervals. CKD = chronic kidney disease; eGFR = estimated glomerular filtration rate; SGLT-2 = sodium-glucose cotransporter-2.

Of the 2,951,404 U.S. individuals eligible by EMPA-KIDNEY, a total of 1,351,147 (1,098,722-1,603,573) individuals had UACR <30 mg/g (45.8%), a total of 830,213 (644,253-1,016,173) had UACR of 30 to 299 mg/g (28.1%), and a total of 770,044 (583,010-957,077) had UACR ≥300 mg/g (26.1%). Of the eligible individuals, 367,756 (272,010-463,502) had eGFR <30 mL/min/1.73 m^2^ (12.5%). The largest Kidney Disease Improving Global Outcomes category of eligible EMPA-KIDNEY individuals was those with A1 albuminuria and G3b eGFR, at 1,184,617 (946,850-1,422,385) individuals (Figure 1).

Compared with EMPA-KIDNEY trial participants, the eligible U.S. population was older (mean age: 71.1 ± 0.6 vs 63.9 in the trial), had higher mean eGFR (44.5 ± 1.0 vs 37.4 mL/min/1.73 m^2^), and had a higher proportion of patients with UACR <30 mg/g (45.8% ± 3.1% vs 20.1%). The U.S. population also had a higher proportion of women (58.1% ± 2.6% vs 33.2%).

Our analysis suggests that over 5 million U.S. individuals with CKD may be eligible for SGLT-2 inhibitors by major trial criteria. EMPA-KIDNEY demonstrated many patients with CKD could benefit from therapy, including those with eGFRs down to 20 mL/min/1.73 m^2^, with our analysis estimating 3 million U.S. individuals eligible. Our analysis also shows up to 367,756 individuals with eGFR <30 mL/min/1.73 m^2^ eligible for empagliflozin in the U.S.—a reminder to clinicians to keep this at-risk population in mind for SGLT-2 inhibitor initiation.

Furthermore, EMPA-KIDNEY established the benefits of SGLT-2 inhibition for patients with lower levels of albuminuria even without diabetes,^1^^,^^5^ building upon what SCORED had shown in patients with diabetes.^4^ This is relevant for national care of CKD patients, as our analysis shows nearly half of U.S. individuals eligible for empagliflozin—at ∼1.4 million adults—had UACR <30 mg/g. This finding supports routine early screening of albuminuria, as EMPA-KIDNEY showed that while benefits were seen across the trial population, empagliflozin’s effect in reducing the risk of progression of CKD was accentuated in those with higher UACR. Thus, earlier identification of at-risk patients could result in population-level improvements in kidney disease. Moreover, despite our results showing that the EMPA-KIDNEY eligible U.S. population was older with higher mean eGFR when compared with the trial population itself, cardiovascular benefits for SGLT-2 inhibition still extended to older patients with higher eGFRs in the SCORED trial—where the median age was 69 years with 47.7% of patients in the sotagliflozin arm with eGFR ≥45 mL/min/1.73 m^2^. This broad generalizability further supports improvements in current gaps in population-level UACR testing to capture the at-risk CKD population who may benefit from risk-lowering therapies.

EMPA-KIDNEY results are broadly generalizable and could benefit approximately 3 million US adults with CKD, with the totality of SGLT-2 inhibitor data supporting use in over 5 million individuals. Routine UACR screening may help identify patients at risk of disease progression likely to benefit from SGLT-2 inhibition.
